# Significant Progress

**Published:** 1896-08

**Authors:** 


### SIGNIFICANT PROGRESS.

LESS than four years ago the U.S.V.M. Association began the agitation for longer courses of instruction in the veterinary colleges of North America. Much was to be said for and against the movement, but the solution of creating a higher standard for its requirements for membership proved a happy and sure settlement of the Association's responsibility.

This step was not taken without some fear and misgivings; but those who so earnestly supported and advocated the movement pledged their faith and belief in its wisdom, and to-day none will gainsay the wisdom of the step.

Many were the predictions and forebodings of the position the schools would take in this matter. Others feared the disintegration of the Association or the creation of another national organization based upon less restrictive requirements for its members. Happily for all concerned, these predictions have not come to pass, and every interest—professional, collegiate, and association—has been conserved and promoted by the courageous position then taken by the U.S.V.M.A.

On the eve of the thirty-third annual gathering it comes before the people of our country stronger, better, and more potent in every channel that has the true interests of the profession at heart. Honored and respected by every college in North America; sustained and aided by every State and local organization throughout the length and breadth of our land; trusted and admired by every member upon whom its privilege of membership has been conferred, well may it convene at Buffalo and take new joy and fresh courage in the work so well performed.

